# Learning strategies and learning styles in distance learning in higher education

**DOI:** 10.3389/fpsyg.2025.1659561

**Published:** 2026-01-12

**Authors:** Emine Kuluşaklı

**Affiliations:** School of Foreign Languages, Malatya Turgut Özal University, Malatya, Türkiye

**Keywords:** distance education, distance learning, E-learning style, higher education, learning strategy

## Abstract

**Introduction:**

The purpose of this study is to identify distance students’ learning strategies and styles and find out the effect of their learning styles on their learning strategies in higher education. It examined 158 faculty survey responses to identify students’ learning strategies (academic thinking, complex cognitive strategy use, management of time and effort, contacts with others, and simple cognitive strategy use) and their learning styles (intuitive, logical, independent, social, active, verbal, and audio-visual) in distance learning.

**Methods:**

A survey study design was used to fully display the learning styles and learning strategies that students employed in distance education.

**Results:**

Research findings displayed that most distance students used a combination of various learning styles and employed all learning strategies at high, medium, or low frequency. The most preferred learning style was the logical learning style and the complex cognitive strategy use was the most preferred learning strategy. In contrast, academic thinking and contacts with others were the least used strategies and audio-visual and independent learning styles were the least preferred styles. The results demonstrated significant relationships between independent learning style and management of time and effort strategy use and verbal, social and audio visual learning styles and contacts with others strategy in distance learning.

**Discussion:**

Explanations for these results and their implications were also discussed within the scope of the study.

## 1 Introduction

Given the very nature of distance learning, distance education ensures chances for learners under different conditions in several geographical places, so learners can access information wherever and whenever they wish. It has been pervaded by the importance of time and the growth of technology. It becomes more vital to explore distance students’ characteristics and to design more efficient distance learning environments (Özonur et al., 2020). In higher education, learning styles have been increasingly important topics influencing various sides of the learning field as they may estimate how learners may solve problems related to learning and process information (Al-Roomy, 2023). As students have various characteristics, their learning styles need to be considered in learning and teaching in higher education. Considering distance students’ need to deal with learning independently, it becomes significant to recognize learning strategies as distance education asks for students to make the right choices and control over their activities (Alliprandini et al., 2015). In distance learning and higher education, students select and implement appropriate learning strategies for effective learning. Therefore, it is vital to choose a learning strategy considering specific situations to reach an educational aim, improve students’ knowledge and reflective use of different learning strategies, and reflect upon the efficiency of these strategies (Zormanová, 2020). Additionally, for academic achievement, students benefit from various types of learning strategies. It is acknowledged that there is a similarity between learning styles and learning strategies as they both are utilized to facilitate the learning process. The correct application of learning strategies concerning students’ learning styles may assist learning (Andrade and Zerbini, 2020). Students’ capacity to select and utilize appropriate strategies, and their awareness of learning styles are prominent elements of their academic achievement (Cabi and Yalcinalp, 2012). Nevertheless, it is not possible to think that a single approach to teaching can work for most of learners or every student. For this reason, being aware of students’ different learning styles can facilitate an efficient learning atmosphere on behalf of learners (Kharb et al., 2013).

Learning strategies are defined as “procedures for acquiring, organizing, or transforming information” (Alexander et al., 1998, as cited in Neroni et al., 2019, p. 1). The roles of learning strategies for various situations have been investigated by many scholars (Akça and Akgün, 2024; Alario-Hoyos et al., 2017; Avila et al., 2021; Hu and Gramling, 2009; Lin et al., 2017; Neroni et al., 2019; Yüksel et al., 2023; Wu et al., 2021; Zhu et al., 2022; Zormanová, 2021). Although students perceive themselves as confident and highly motivated to achieve in the course, learning strategies need to be developed concerning time management (Alario-Hoyos et al., 2017). The study results indicate a positive relationship between distance education students’ academic performance and the complex strategy use and the management of time and effort. Specifically, students obtain the lowest score on contact with others and the highest score on complex cognitive strategy use. Students’ achievement and learning experiences in distance learning are influenced by the use of cognitive learning strategies (Neroni et al., 2019). Similarly, students report that they use complex cognitive strategies most frequently in learning strategies in distance education. However, communication with others is the least used strategy. The most important complex cognitive strategy in the process of distance learning is the cognitive strategy use. Learners in online learning do their best to gain self-regulation and academic success (Yüksel et al., 2023). It is highlighted that including cognitive learning strategies in distance education plays an essential role in improving students’ learning and performance. In distance education, providing courses and monitoring students to improve their learning strategies can contribute to students’ success (Akça and Akgün, 2024). Learning strategies affect online learning satisfaction of learners by means of academic emotions (Wu et al., 2021). It is recommended that students’ final grades, perceived progress, and satisfaction be increased by using an online learning strategy. Instructors teaching in distance education should support their students’ self-regulation skills (Lin et al., 2017). It is asserted that distance education students prefer some strategies most including “paraphrasing, summarizing, underlining/highlighting key information in the text, categorizing information to remember the text better, writing notes, creating analogies, as well as generative note- taking, question answering, retelling of the text in their own words, and preparation of answers to memorize key information better” (Zormanová, 2021, p. 12). In another study, MOOC students utilize self-management strategies containing resource management and time management, self-monitoring including authentic tasks, self-reflection etc., and task strategies comprising watching videos, taking notes (Zhu et al., 2022). Students state that cognitive, time, and effort management/goal setting are the most effective strategies to be successful in online courses (Hu and Gramling, 2009). Freshmen students use rehearsal strategies most followed by elaboration, organization skills, time and study strategies, peer learning, critical thinking, and self-regulation and effort regulation (Avila et al., 2021).

Learning style is “an individual’s preferred way of learning” (Santo, 2006, p. 71). Kolb’s classification for learning styles covers accommodator, convergent, assimilator, and divergent styles. According to Kolb, among the four learning styles, the accommodator learning style is the most suitable one for distance learning (as cited in Schultz and Schultz, 2004). These learners like trying innovative conditions and applying plans and course materials to solve real problems (Conner, 2003, as cited in Schultz and Schultz, 2004). There are four groups of learning styles in the context of online learning. These include problem-based, social, perceptual, and cognitive processing. Students enjoy practicing problem-solving skills in problem-based learning styles. Personality traits are taken into consideration for social learning styles. Learners focus on a specific perceptual sense as auditory, visual, textual, or active learning in this type of learning style. Cognitive processing learning styles contain analytic, global or holistic, random, and serial learners (Alalshaikh, 2015). Additionally, learners taking distance learning courses are separated into independent learners and collaborative and participant learners. While participants and collaborative students prefer to study collaboratively, independent students prefer studying on their own (Logan and Thomas, 2002). For Fuhrmann and Grasha (1983), six learning styles are competitive, in which learners want to do better than other learners, collaborative including learners appreciate group work and working well with other learners, dependent in which learners demand help and detailed instructions, independent in which learners need less help and prefer to study independently and avoidant including learners waiting for the last minute and doing less work (as cited in Santo, 2006). According to Honey and Mumford, there are mainly four learning styles: pragmatics, theorists, reflectors, and activists (as cited in Santo, 2006). The index of learning styles includes “information processing: Active and Reflective learners, information perception: Sensing and Intuitive learners, information reception: Visual and Verbal learners and information understanding: Sequential and Global learners” (Klašnja-Milićević et al., 2011, p. 889).

Effective learning in distance education can be developed by having adequate knowledge of learning styles and respect for individual differences, as new technologies have been growing in the world. However, there is not enough attention given to individual differences, although distance learning serves as an alternative for most adult students (James and Gardner, 1995). It can sometimes be hard for instructors to teach at distance education to realize the significance of students’ needs and be more involved with the mechanics of course delivery than students’ demands (Richmond and Cummings, 2005). The investigation of individual differences and learning activities in education and the importance given to these differences have been under debate openly recently, so learning style preferences should not be avoided (Özonur et al., 2020). It is stated that the influence of learning styles is disregarded by some scholars and if students’ preferences in learning and teaching are not taken into account, significant problems can occur such as poor academic achievement and retention of students in courses. Therefore, it is necessary to consider learning styles as distance learning includes various students’ characteristics (Wanna and de Jesus Simões, 2021). Similarly, institutions and instructors need to determine the learner characteristics of successful online students since online courses have been growing rapidly (Zacharis, 2011). Learning styles of students have a significant effect on online participation (Cheng and Chau, 2016) as they affect students’ capacity to join an online course successfully (Santo, 2006). However, some research indicates no connection between learning styles and satisfaction with course format (Wu, 2014).

Some research focuses on the link between learning styles and strategies used in distance learning (Balci, 2017; Battalio, 2009; Feng et al., 2019; Hajar, 2014; Jie and Xiaoqing, 2006; Kia et al., 2009; Mangwende, 2024; Manochehr, 2006; Mekie and Tefera, 2021; Özonur et al., 2020; Tabanlioglu, 2003). Students accommodated in various geographical locations have an opportunity to access information free from the place and time in distance learning. For this reason, they take responsibility for their own learning and must select an independent learning style. It is stated that the independent learning style is the most preferred style among students studying at two distance education programs at a university. It is followed by the verbal one. Besides, the link between students’ learning styles and the educational model can enhance students’ academic success (Özonur et al., 2020). In another study, the off-campus undergraduate students enrolled in a distance education program preferred the independent learning styles most and the collaborative learning styles the least. Although independent learners think they are responsible for their learning, collaborative learners share their ideas in groups. It is asserted that students have limited opportunities and time to interact with their peers and instructors due to their responsibility in daily life; therefore, they lack collaboration (Hajar, 2014). It was acknowledged that learners who learned best through observations, field work, and laboratories, namely converger learners, and who learned best through analogies, papers, and lectures, namely assimilator learners, were better at learning with web-based or e-learning (Manochehr, 2006). Reflective learners benefit most from the online environment and are more successful academically than active learners, while sequential learners are more successful than global learners (Battalio, 2009). Similarly, it is asserted that visual students are more successful than those with solitary, verbal, aural, and social learning styles. Nevertheless, physical and logical students have the least academic achievement in distance education (Kia et al., 2009). Instructors try to accommodate students’ learning styles in distance education by giving numerous assessment forms, making materials, and using different platforms. However, they meet difficulties such as learners’ demotivation, workload, and technical problems (Maryono and Lengkanawati, 2022). To help distance students use the available learning resources, online seminars, and workshops become vital (Mangwende, 2024). There is a distinction between learning strategies and learning styles as “learning styles embody unconscious individual learner traits while learning strategies are specific behaviors selected by the learner to make learning more efficient” (Jie and Xiaoqing, 2006, p. 68). The correlation between learning strategies and styles is explored by educators. It is confirmed that all strategies except the affective ones are related to the pragmatic learning style, and metacognition, cognition, and memory strategies are related to the theoretical learning style. Besides, there are links between cognition strategies and the reflective learning style and between social, compensation, and memory strategies and the active learning style. It is suggested that learning styles be examined and learning strategies be given priority and increase their positive effects in the area of foreign language learning (Feng et al., 2019). Similarly, the positive relationship between learners’ language learning strategy and their learning style preferences is found to be significant as affective, metacognitive, compensation, cognitive, and memory strategies have positive correlations with auditory and visual learning styles, and these strategies, except affective strategies, are positively related to the kinesthetic learning style. It is claimed that students’ learning styles significantly influence language learning strategy preferences (Balci, 2017). Besides, a positive link is observed between visual learning style and cognitive strategy use, and a negative correlation is found between compensation strategy use and tactile learning style in English language learning (Mekie and Tefera, 2021). Affective strategies are shown to have a significant relation with visual learning styles, and social, affective, memory, and cognitive strategies have significant correlations with auditory learning styles. Additionally, compensation strategies are closely related to individual learning styles. However, there are no statistically significant differences between metacognitive strategies and any learning styles (Tabanlioglu, 2003). It is proposed that learning styles have an important impact on students’ learning strategy preferences and their learning outcomes (Jie and Xiaoqing, 2006).

To date, numerous studies have attempted to understand students’ learning strategies and learning styles and relate them to higher education. However, most of this research has been carried out with on-campus university students having traditional face-to-face education. Moreover, much research deals with only students’ learning strategies or styles according to the variables, such as academic achievement, mainly in the English as a foreign language context. However, less is known about learning strategies and styles in the context of distance education (Zormanová, 2020) and the relationship between distance learners’ learning styles and strategies, while the number of learners, distance education courses, and the trend are increasing worldwide. For lifelong learning, learning strategies are also essential as information and technology are constantly growing and changing in today’s world and there has been a gradually increasing number of online learners with the increase of distance education worldwide. How students in distance education study in online courses, which learning strategies they use to reach their learning outcomes, and the learning styles of students in online environments are studied by many researchers. Although various studies focus on students’ learning strategies and learning styles in traditional face-to-face education, to the best knowledge of the present author no research has been carried out to identify the correlation between learning strategies and learning styles in the domain of distance education. No single study has investigated to identify both learning strategies and learning styles of distance education students, as far as the literature review in this study is concerned, as supported by the result of the study, indicating that there are not sufficient studies on learning strategies, including adult learners in the distance education context (Filcher and Miller, 2000).

Learning styles and strategies can practically improve online learning environments if instructors comprehend students’ way of learning and processing information and perception students can be successful in online learning (Vyjayanthi, 2015). So, it is assumed that learning styles and strategies can practically improve online learning environments as “individuals learn better when they receive information in their preferred learning style” (Dekker et al., 2012, as cited in Hattie and O’Leary, 2025, p. 5), and if learners use metacognitive and cognitive strategies, learning becomes more efficient (Hattie and O’Leary, 2025). Research of this type can shed new light on the present learning and teaching situation in the domain of distance education and higher education. Therefore, this study fills the gap in the existing research on identifying learning strategies and styles in distance education in higher education, as there have not been any studies focusing on exploring distance learners’ learning style and strategy preferences and the correlation between these. For this reason, the study purpose is to identify distance students’ learning strategies and styles and find out the effect of their learning styles on their learning strategies in distance higher education as indicated in the research questions below:

RQ1: What are the learning strategies of the students in distance education?

RQ2: What are the learning styles of the students in distance education?

RQ3: How do learning styles influence the use of learning strategies of the students in distance education?

## 2 Materials and methods

### 2.1 Research design

The survey method was used to display an overall picture of the learning strategy and learning style distributions of the distance education students and the relationship between learning styles and learning strategies in higher education. This study was conducted by using the quantitative method of descriptive research.

### 2.2 Sample and data collection

This study comprised 158 distance students including 95 females and 63 males attending formal education at four faculties and three vocational schools at a public university. Data were collected by conducting an online questionnaire with students who attended online courses during the academic year 2024–2025. The questionnaires were administered to students by using a random sampling method. The students were recruited from a public university in Türkiye. Participants accessed the questionnaires through an anonymous hyperlink online. They were informed about the terms of participation and the aim of the study at the beginning of the questionnaire. They gave consent to the terms to display that they were willing to participate in the study voluntarily. The study was approved by the Research Ethics Committee of the university where the present research was conducted. It took students about 10–15 min to complete two questionnaires.

### 2.3 Research tools

#### 2.3.1 Learning strategies

The Scale of Learning Strategies for Distance Education Students, developed by Meijs et al. (2019) and adapted to Turkish by Geçer and Deveci Topal (2023), was used in the research to determine students’ distance education learning strategies. The scale was constructed as a 7-point Likert-type and consisted of 23 items. It has five sub-categories such as simple cognitive strategy use (five items), academic thinking (five items), management of time and effort (four items), contacts with others (four items), and complex cognitive strategy use (five items). In the collected data a higher score displayed the stated learning strategy was used more effectively. The means of the subscales were used to determine which learning strategies were utilized in the context of distance education. The Cronbach alpha coefficient obtained for the reliability of the study for the overall scale was calculated as 0.915 for the original version. Additionally, Cronbach alpha values were calculated as 0.88 for the academic thinking, 0.76 for the contacts with others, 0.87 for the simple cognitive strategy use, 0.82 for the complex cognitive strategy use, and 0.69 for the management of time and effort.

#### 2.3.2 Learning styles

The e-Learning Styles Scale for Electronic Environments was developed by Gülbahar and Alper (2014) to identify students’ learning styles in online environments. It was developed with the learners enrolled in a distance learning program at a university. It comprised seven factors containing 38 items. These included intuitive learning styles (four items), logical learning (three items), independent learning (four items), social learning (six items), active learning (six items), verbal learning (seven items), and audio-visual learning (eight items). It was a 5-point Likert-type scale. The Cronbach alpha coefficient obtained for the reliability of the study for the overall scale was calculated as 0.94 for the original version. The Cronbach alpha values were 0.82 for independent learning, 0.87 for social learning, 0.86 for audio- visual, 0.83 for active learning, 0.86 for verbal learning, 0.77 for logical learning, and 0.72 for intuitive learning.

### 2.4 Data analysis methods

For data analysis, groups were formed by using the mean, mode, median, and standard deviation on the learning strategies and learning styles scales to identify the dominant learning strategies and learning styles of such students in distance education. Descriptive statistics as standard deviations (Sd stands for standard deviation) and means (M stands for mean) for the factors of the scales, were given as tables in the results section. The normality of the data was analyzed using skewness and kurtosis measures and the Kolmogorov–Smirnov test. It was found that the data were normally distributed, as the skewness and kurtosis values identifying students’ learning strategies and learning styles were within the acceptable range and the Kolmogorov–Smirnov values were higher than 0.05 for each factor. The statistical procedure of Pearson correlation and regression analysis was the analysis to investigate the relationship between learning strategies and learning styles of students in distance education. The SPSS Statistics for Windows Program was used for data analysis, through which the reliability of the questionnaires was calculated.

## 3 Results

The results of the data analysis were given in this part. The present study was conducted with a limited sample size and relied on participants’ self-reporting.

### 3.1 Descriptive statistics for learning strategies

To understand the learning strategies used by distance education students, the Learning Strategies for Distance Education Students Scale (Geçer and Deveci Topal, 2023) items were subjected to descriptive analysis according to five factors. The five factors were represented by the complex cognitive strategy use, the simple cognitive strategy use, the management of time and effort, the academic thinking, and the contacts with others. Cognitive strategies can be “directly applicable to a certain task or course (e.g., rehearsal, organization)” (Alexander et al., 1998 as cited in Neroni et al., 2019, p. 1) referring to complex cognitive strategy use (e.g., I try to think through a topic and decide what I am supposed to learn from it rather than just reading it over when studying for this course) and simple cognitive strategy use (e.g., when I study for this course, I write brief summaries of the main ideas from the readings and my course notes). Management of time and effort refers to “part of behavior control, which is necessary in the academic learning domain” (Pintrich, 2004 as cited in Meijs et al., 2019, p. 7) (e.g., I attend this course regularly), academic thinking refers to “a critical attitude regarding the course material and on the use of the material as a starting point for interweaving the information with previous and common knowledge” (Meijs et al., 2019, p. 7) (e.g., I often find myself questioning things I hear or read in this course to decide if I find them convincing) and contacts with others is related to peer learning and help seeking (Neroni et al., 2019) (e.g., I ask the instructor to clarify concepts I do not understand well). Table 1 provides summary statistics of the students’ ratings. It includes the mean, mode, median, and standard deviation for each learning strategy use for distance education.

**Table 1:** Descriptive statistics and reliability values.

Variable	Mean (M)	Standard deviation (Sd)	Cronbach’s alpha
Complex cognitive strategy use	5.40	1.26	0.83
Simple cognitive strategy use	5.20	1.38	0.86
Management of time and effort	4.99	1.25	0.76
Academic thinking	4.77	1.40	0.85
Contact with others	4.25	1.50	0.79
Overall	4.92	1.35	0.93

As shown in Table 1, the overall mean score of learning strategy use in distance education was 4.92, indicating that students partly employed learning strategies effectively. Specifically, complex cognitive strategy use (*M* = 5.41; Sd = 1.26) and simple strategy use (*M* = 5.20; Sd = 1.38) had the highest mean scores, displaying that students used these strategies effectively. Time and effort management (*M* = 4.99; Sd = 1.25) and academic thinking (*M* = 4.77; Sd = 1.40) were the learning strategies that students partly employed these strategies effectively. The contacts with others was found to be the least used strategy as compared to others (*M* = 4.25; Sd = 1.50), indicating that students partly utilized this strategy in an efficient way.

### 3.2 Descriptive statistics for learning styles

The e-Learning Styles Scale for Electronic Environments (Gülbahar and Alper, 2014) was subjected to descriptive analysis according to seven factors to understand the learning styles of distance students. Seven learning styles were represented by logical, intuitive, active, verbal, social, and audio-visual. Logical learning styles refer to “study step by step following a plan and best learn by reflection” (Özonur et al., 2020, p. 1860) (e.g., mathematics, science and technology are my favorite subjects), and intuitive learning styles refer to “more interested in overviews and a broad knowledge (bored with details)” (Klašnja-Milićević et al., 2011, p. 889) (e.g., I prefer random flows rather than step-by-step and hierarchical processes). Active learning styles are related to “try out new material immediately” (Klašnja-Milićević et al., 2011, p. 889) (e.g., I love learning through playing games and simulations), and verbal learning styles refer “to perceive materials as texts” (Klašnja-Milićević et al., 2011, p. 889) (e.g., I have a very wide vocabulary and I like to use the right word in the right place). Social learning styles refer to “give importance to interaction in their learning process and prefer to participate in activities and projects that require group work” (Özonur et al., 2020, p. 1860) (e.g., while learning with the e-learning method, I like to do group work interactively with other students), and independent learning styles refer to “study on their own and take responsibility for their learning” (Özonur et al., 2020, p. 1860) (e.g., I am quite confident in my ability to learn on my own). Audio-visual learning styles refer to “best learn by hearing and seeing and favor teachers who explain subjects in detail” (Özonur et al., 2020, p. 1860) (e.g., I easily remember visual objects, plans, and situations). The ranking of learning styles in distance education in terms of seven factors is demonstrated in Table 2.

**Table 2:** Descriptive statistics and reliability values.

Variables	Mean (M)	Standard deviation (Sd)	Cronbach’s alpha
Logical learning style	2.84	1.07	0.68
Intuitive learning style	2.69	0.85	0.56
Active learning style	2.67	0.84	0.63
Verbal learning style	2.64	0.79	0.78
Social learning style	2.56	0.93	0.82
Independent learning style	2.20	1.05	0.86
Audio-visual learning style	2.16	0.87	0.84
Overall	2.53	0.91	0.92

As Table 2 displayed, the overall mean score of students’ learning styles in distance education was determined to be at an average level (*M* = 2.53; Sd = 0.91). In detail, logical learning styles had the highest mean score (*M* = 2.84; Sd = 1.07). Intuitive (*M* = 2.69; Sd = 0.85), active (*M* = 2.67; Sd = 0.84), verbal (*M* = 2.64; Sd = 0.79), and social (*M* = 2.56; Sd = 0.93) were the learning styles used at a medium level, respectively. The levels of independent (*M* = 2.20; Sd = 1.05) and audio-visual learning styles (*M* = 2.16; Sd = 0.87) were determined to be the least preferred strategies as compared to others.

### 3.3 The relationship between learning styles and learning strategies

For the study in which the relationship between students’ learning styles and learning strategies was examined, the Pearson Correlation Test was administered as the data displayed a normal distribution, as shown in Table 3.

**Table 3:** Pearson correlations.

	Variable	Mean	Sd	Learning strategy	Learning style
1	Learning strategy	4.95	1.08	–	0.197*
2	Learning style	2.50	0.65	0.197*	–

*Correlation is significant at the 0.05 level (two-tailed).

The results of Pearson correlation analysis displayed that learning styles were significantly correlated with learning strategies (*p* < 0.05).

To understand the relative importance of each independent variable, namely intuitive, logical, independent, social, active, verbal, and audio-visual learning styles in predicting learning strategy use, regression analysis is administered as indicated in the following tables.

Table 4 shows that independent variables, namely intuitive, logical, independent, social, active, verbal, and audio-visual learning styles have explained 021% of the variance regarding time management strategy. No significant differences have been found between time management strategy and audio-visual, verbal, active, social, logical, and intuitive learning styles (*R* = 0.255; *R*2 = 0.021; *F* = 1.490; *p* > 0.05). However, a significant difference has been obtained between independent learning style and time management strategy.

**Table 4:** Regression analysis of learning strategies sub-categories and time management.

Unstandardized coefficients		Standardized coefficients		Collinetary statistics	
Model	*B*	Std. error	Beta	*t*	Sig
(Constant)	5.368	0.456		11.785	0.000
Audio visual	0.057	0.192	0.040	0.300	0.765
Verbal	−0.088	0.179	−0.055	−0.491	0.624
Active	0.093	0.154	0.062	0.601	0.549
Social	−0.150	0.134	−0.112	−1.119	0.265
Independent	−0.298	0.145	−0.250	−2.056	0.042
Logical	0.000	0.106	0.000	0.003	0.998
Intuitive	0.195	0.140	0.133	1.388	0.167

*R* = 0.255, adjusted *R*2 = 0.021, *F* = 1.490.

Table 5 indicates that independent variables namely intuitive, logical, independent, social, active, verbal, and audio-visual learning styles have explained 05% of the variance regarding complex strategy use. No significant differences have been obtained between complex strategy use and intuitive, logical, independent, social, active, verbal, and audio-visual learning styles (*R* = 0.304; *R*2 = 0.050; *F* = 2.188; *p* > 0.05).

**Table 5:** Regression analysis of learning strategies sub-categories and complex strategy use.

Unstandardized coefficients		Standardized coefficients		Collinetary statistics	
Model	*B*	Std. error	Beta	*t*	Sig
(Constant)	6.717	0.451		14.909	0.000
Audio visual	0.082	0.190	0.056	0.430	0.668
Verbal	−0.214	0.177	−0.134	−1.208	0.229
Active	0.174	0.153	0.116	1.140	0.256
Social	−0.169	0.133	−0.125	−1.269	0.206
Independent	−0.156	0.143	−0.131	−1.090	0.277
Logical	−0.078	0.105	−0.066	−0.742	0.460
Intuitive	−0.144	0.139	−0.098	−1.035	0.302

*R* = 0.304, adjusted *R*2 = 0.050, *F* = 2.188.

Table 6 displays that independent variables namely intuitive, logical, independent, social, active, verbal, and audio-visual learning styles have explained 021% of the variance regarding simple strategy use. No significant differences have been obtained between simple strategy use and intuitive, logical, independent, social, active, verbal, and audio-visual learning styles (*R* = 0.255; *R*2 = 0.021; *F* = 1.487; *p* > 0.05).

**Table 6:** Regression analysis of learning strategies sub-categories and simple strategy use.

Unstandardized coefficients		Standardized coefficients		Collinetary statistics	
Model	*B*	Std. error	Beta	t	Sig
(Constant)	6.072	0.502		12.100	0.000
Audio visual	0.204	0.211	0.128	0.964	0.337
Verbal	−0.287	0.198	−0.164	−1.450	0.149
Active	0.191	0.170	0.116	1.125	0.263
Social	−0.004	0.148	−0.003	−0.030	0.976
Independent	−0.166	0.160	−0.126	−1.038	0.301
Logical	−0.166	0.117	−0.128	−1.417	0.159
Intuitive	−0.080	0.155	−0.050	−0.518	0.606

*R* = 0.255, adjusted *R*2 = 0.021, *F* = 1.487.

Table 7 indicates that independent variables namely intuitive, logical, independent, social, active, verbal, and audio-visual learning styles have explained 112% of the variance regarding contacts with others strategy. It can be said that audio visual, verbal, and social learning styles have had a positive effect on contacts with others strategy (*R* = 389; *R*2 = 112; *F* = 3.828; *p* < 0.05). According to the standardized regression coefficients, the relative importance of the predictor variables on contacts with others has been as follows: Audio visual (*β* = 0.444), social (*β* = 0.368), and verbal (*β* = 0.291).

**Table 7:** Regression analysis of learning strategies sub-categories and contacts with others.

Unstandardized coefficients		Standardized coefficients		Collinetary statistics	
Model	*B*	Std. error	Beta	*t*	Sig
(Constant)	5.304	0.520		10.195	0.000
Audio visual	0.768	0.219	0.444	3.505	0.001
Verbal	−0.554	0.205	0.291	2.704	0.008
Active	0.128	0.176	0.072	0.729	0.467
Social	−0.593	0.153	0.368	3.866	0.000
Independent	−0.075	0.166	−0.052	−0.452	0.652
Logical	0.102	0.121	0.073	0.845	0.400
Intuitive	−0.075	0.160	−0.043	−0.469	0.640

*R* = 0.389, adjusted R2 = 112, *F* = 3.828.

Table 8 displays that independent variables, namely intuitive, logical, independent, social, active, verbal, and audio-visual learning styles have explained 033% of the variance regarding academic thinking strategy. No significant differences have been obtained between academic thinking strategy and intuitive, logical, independent, social, active, verbal, and audio-visual learning styles (*R* = 0.275; *R*2 = 0.033; *F* = 1.756; *p* ˃ 0.05).

**Table 8:** Regression analysis of learning strategies sub-categories and academic thinking.

Unstandardized coefficients		Standardized coefficients		Collinetary statistics	
Model	*B*	Std. error	Beta	*t*	Sig
(Constant)	6.031	0.505		11.949	0.000
Audio visual	0.397	0.212	0.247	1.866	0.064
Verbal	–0.344	0.199	–0.194	−1.732	0.085
Active	–0.111	0.171	–0.066	–0.646	0.519
Social	–0.144	0.149	–0.096	–0.970	0.333
Independent	–0.101	0.161	–0.076	–0.630	0.530
Logical	0.102	0.118	0.078	0.870	0.386
Intuitive	–0.227	0.156	–0.139	−1.458	0.147

*R* = 0.275, adjusted *R*2 = 033, *F* = 1.756.

## 4 Discussion

Considering the first research question, the learning strategies that characterized students in distance education were investigated. When analyzing which learning strategies characterized students in distance education, the findings indicated that overall learning strategy use in distance education was at an average level. In detail, the complex cognitive strategy use was found to be the most preferred learning strategy the result of which was in line with the previous studies indicating that the most frequently used learning strategy for distance learning students was complex cognitive strategy use (Neroni et al., 2019; Yüksel et al., 2023) and cognitive strategies were the most useful strategies for students to achieve in online courses (Hu and Gramling, 2009; Yüksel et al., 2023). The reason behind this preference might be related to the idea that students preferred complex cognitive strategy at a higher level than other learning strategies since distance education required learners to have more responsibility than traditional on-campus education (Yüksel et al., 2023). The study’s results indicated that students tried to think through a topic and decide what they were supposed to learn from it rather than just reading it over when studying for the course. Besides, they tried to relate the material to what they already knew when reading for the course, they memorized keywords to remind them of important concepts, and when studying for this course, they tried to determine which concepts they did not understand well (Meijs et al., 2019). It was displayed that complex cognitive strategy use could be a positive predictor of academic performance (Yüksel et al., 2023). Therefore, to enhance learners’ performance and learning in distance education, the cognitive strategy use was a necessary tool (Akça and Akgün, 2024). Besides, the simple cognitive strategy was the second most preferred strategy by students. In detail, when students studied for the course, they went over their course notes, made an outline of important concepts and lists of important items, and memorized the lists. They read their course notes and the course readings over and over again, and wrote brief summaries of the main ideas from the readings and their course notes (Meijs et al., 2019). Cognitive strategies in distance learning were important instruments used by students most to improve their learning since these strategies were commonly utilized, providing learners with enjoyable, challenging, and interesting classroom activities in learning. They also helped learners understand the material more easily (Hidayatullah and Dayu, 2017). For this reason, they were preferred by students.

Regarding the management of time and effort strategy, the findings indicated that it explains only 2.1% of variance, as students were not successful enough to manage to keep working until they finished when the course materials were dull and uninteresting. Besides, they were not sure that they kept up with the weekly readings and assignments, and they did not make good use of their study time for the course adequately (Meijs et al., 2019). This result might be related to the idea that distance learners were often busy in their family and work life so they needed to have more time management skills in order to be academically successful (Neroni et al., 2019). However, it is one of the self-regulated skills containing planning, programming, and managing personal study time, and is also an important aspect to be enhanced by students. To make students manage their time effectively, courses can include balanced weekly content, so students may follow a routine knowing the weekly workload for each assignment. Therefore, time management strategies should be improved as previously indicated in the research (Alario-Hoyos et al., 2017). Furthermore, in terms of using academic thinking strategy, students lacked using necessary academic thinking strategy, which was related to “a critical attitude regarding the course material and on the use of the material as a starting point for interweaving the information with previous and common knowledge” (Meijs et al., 2019, p. 7). That is to say, students were not good enough to make up questions to help focus their reading and lacked questioning things they heard or read in the course to decide if they found them convincing when reading for the course. It was stated that there was a need for academic thinking to think and work scientifically (Meijs et al., 2019).

For the contacts with others strategy use, students scored the lowest among other factors of the learning strategy scale, the result of which was in line with the earlier research indicating that it was the least preferred strategy used by distance learners (Neroni et al., 2019; Yüksel et al., 2023). Although students asked the instructor to clarify concepts they did not understand well and tried to identify students in the course whom they could ask for help if necessary, they did not try adequately to work with other students from the course to complete the course assignments and when studying for the course and often set aside time to discuss the course material with a group of students from the course (Meijs et al., 2019). The reason behind these findings might be that distance students did not have enough time and chances to work or interact with their instructors and classmates, as they also had to continue their daily life (Hajar, 2014). Besides, in the distance education setting, interaction with the course instructor and the peers was limited. However, interacting with peers and instructors had a vital role for successful academic performance as students’ grades increased when their interaction with instructors and students about the course material was getting more (Neroni et al., 2019). It is essential to support students’ active participation and make connections in online learning. Students need a more inclusive and interactive learning atmosphere. If students’ confidence is supported through a suitable learning environment, their level of satisfaction with online learning may increase. Verbal communication during online courses is one of the tools that can be utilized for student engagement (El Moussaddar et al., 2025).

Concerning the second research question, students’ learning styles were explored. The results showed that the logical learning style was the most preferred one, followed by intuitive, active, verbal, social, independent, and audio-visual learning styles, respectively, in distance education. They indicated that the most preferred learning style of distance students was the logical learning style, the result of which did not corroborate the findings of the previous studies on distance learners’ learning styles, indicating that the most preferred learning style among distance students was the independent learning style (Gülbahar and Alper, 2014; Hajar, 2014; Özonur et al., 2020). The fact that students preferred the logical learning style the most displayed that mathematics, science, and technology were their favorite subjects and they liked dealing with operations that required calculations. This result could be related to the participating students’ majors, as some were studying at various departments such as engineering, economics etc. Additionally, they enjoyed playing logical games like chess and solving puzzles as indicated in the previous study that logical students learned best by thinking in detail and liked to play logical games and solve puzzles (Gülbahar and Alper, 2014). It is stated that field-independent learners tend to be more logical and analytical and they are good at restructuring and abstracting ways of a problem. They are less social and academically successful in an impersonal learning environment (Lu et al., 2003).

With regard to the intuitive learning style, students preferred random flows rather than step- by-step and hierarchical processes as intuitive learners were inclined “to perceive the possibilities that arise from a situation, rather than just the hard facts revealed” (Jie and Xiaoqing, 2006, p. 81). They did not like their learning process to be planned by others they used their intuition when solving problems and they learned best by associating with their emotions. It was probably because intuitive learners were very creative and learned best by connecting with their feelings. They used their intuition to solve problems (Gülbahar and Alper, 2014). Regarding the active learning style, students could play a musical instrument or sing, and they loved doing handicrafts like ceramics and sculpture. It was stated that active students liked searching, investigating, and learning through games and learned best by experiencing (Özonur et al., 2020). The study results demonstrated that verbal students loved telling jokes and stories, had a very wide vocabulary, liked to use the right word in the right place, and they thought they learned best by reading. They liked subjects such as foreign languages, history, and literature. They considered they learned better by reading and liked discussions (Özonur et al., 2020). In terms of social learning styles, students liked doing group work with other students interactively, they preferred activities and projects that required group work, they liked to participate in asynchronous activities (forum, blog, wiki, etc.) while learning with the e-learning method. Social students liked to participate in group discussions, projects, and activities and consider learning as the responsibility of learners and teachers (Gülbahar and Alper, 2014). Instructors could use interactive group activities and projects that require group work, encourage students’ participation in synchronous activities such as chat, virtual classroom, and whiteboard applications, and students’ interaction with their peers and the instructor (Gülbahar and Alper, 2014).

Considering the independent learning style, the previous study asserted that independent students took responsibility for their own learning and liked to work on their own (Özonur et al., 2020). In line with this idea, for the independent learning style, students had moderate confidence in their ability to learn on their own and preferred to work independently with guidance. Finally, the results displayed that the audio-visual learning style was the least preferred one by students in distance education. It was displayed that the audio-visual students preferred documents including cartoons, paintings, and images, and were more interested in searching and discovering. They best learned by seeing and hearing, and liked teachers who explained subjects in detail (Gülbahar and Alper, 2014). Nevertheless, students in this study did not like books with a lot of figures, cartoons, and tables. They did not enjoy hearing people share their different experiences much and they could not learn best by seeing. Additionally, they could not easily remember visual objects, plans, and situations. In order to enhance audio-visual learners’ participation in classes, instructors can design activities for audio-visual learners. They can make written documents and handouts as visually appealing as possible, organize visual presentations in a better way and use visual materials in different formats (Lobas and Serdega, 2020). Visual objects, music, songs, charts, pictures, caricatures, and videos can be used for these learners. Moreover, a video component and integration of various media tools into a course provide students the chance to employ media most suitable to individual learning preferences and styles (Rajesekaran and Rajesekar, 2015).

The third research question elicited about how students’ learning styles affected the learning strategy use in distance education. By looking at the study’s results, it was clear that to some extent, there was a relationship between the learning style and strategy use of students as they frequently applied strategies that represented their style preferences (Sahragard et al., 2014). This finding was consistent with the previous research displaying the impact of students’ learning styles on their learning strategy preferences (Balci, 2017; Jie and Xiaoqing, 2006). Further analyses of the results indicated that a particular learning style was related to the learning strategies that “fall into their own types” (Jie and Xiaoqing, 2006, p. 79). The independent learning style was found to significantly influence the management of time and effort strategy. In detail, independent learners could manage their time well as they had the capacity to take responsibility for their learning (Özonur et al., 2020) and they were confident enough as they believed they could manage their learning on their own. In distance education, students were required to be self-regulated learners and make more effort to be successful since this type of education offered flexibility both in time and place. The independent learners could use their study time well, regularly attend the course, and try to finish the course materials, whether they were uninteresting. Moreover, the results displayed that verbal, audio-visual, and social learning styles were associated with the contacts with others strategy. In other words, the contacts with others strategy was the strategy mostly chosen by students with various learning styles as verbal, social, and audio-visual. This strategy included such activities as studying with other students and help-seeking from other students. Social learners, with their preferences for talking and action, would naturally show a strong liking for this strategy. Learners who were interested in working in groups usually had more communication and interaction with others (Sahragard et al., 2014). Moreover, audio- visual learners liked hearing people share their different experiences and preferred to use the contacts with others strategy, prioritizing interaction with others. They preferred instructors who explained the subjects in detail in courses and they were very realistic. With regard to verbal learners, it was stated that verbal learning could force learners to choose a specific learning style to perform better in school and get more information (Candilas et al., 2023). Verbal learners tend to learn by listening, reading, or in words and writing. The contacts with others strategy, which concerned peer learning and help-seeking (Neroni et al., 2019), was preferred by verbal learners in this research.

Although students with a particular learning style preferred at least one learning strategy (Jie and Xiaoqing, 2006), the results of the current study indicated that students with active, logical, and intuitive learning styles did not tend to use any specific learning strategies.

If educators know students’ learning styles, they can comprehend why students had difficulty understanding or did not perform well in a specific activity (Vyjayanthi, 2015). There are some actionable steps educators can take to apply the study findings such as applying active learning strategies to improve learning and providing help for students take responsibility for their learning by being a guide rather than an expert authority. They can determine students’ demands and needs and then apply strategies by giving feedback on their learning outcomes, so students feel more satisfied and comfortable in the distance learning environment. Therefore, using online strategies can result in accommodating lifelong learning (Phillips, 2005). It is suggested that it would be useful to track how student strategies change throughout a semester or program for future research.

## 5 Conclusion

The current study addressed the gap in the literature identifying the learning styles and learning strategies of students in distance learning in higher education, and explored the predictive value of students’ learning styles on their learning strategy use in distance education. The results presented here allowed us to answer three research questions and have implications for understanding learners’ learning strategies and learning styles in distance education and other similar learning environments. Concerning distance students’ learning strategy preferences, the research findings showed that students tended to employ all learning strategies, preferring the complex strategy most and contacts with others least. This result indicated that it could be beneficial to allow students to practice and teach complex cognitive strategies in distance courses for academic achievement, since the complex cognitive strategy use was regarded as a positive indicator of academic performance (Neroni et al., 2019). Learning strategies were evenly distributed among students in distance education. Generally, students did not have a clear preference for any strategies, and when they took online courses, they used a combination of many learning strategies. With regard to learning styles, students had various learning styles. Most students had logical learning styles while audio-visual and independent learners were less in distance education. The independent learning style led to the management of time and effort strategy while verbal, social, and audio-visual learning styles led to the contacts with others strategy.

This study will contribute to the field of higher education and distance education in many ways. It contributes to understand the correlation between learning strategies and learning styles in distance learning in higher education. It may enhance instructors’ awareness of their teaching and learning strategies and styles. Besides, it may help students raise their awareness concerning their learning strategy preferences and styles. Distance learners may be aware of their strengths and weaknesses in their learning process if educators identify preferred learning styles and learning strategies of students, plan and design learning activities, respectively. Understanding students’ learning styles can help them improve their strategy preferences and achieve academic goals in learning. Moreover, it was suggested learning styles be incorporated into learning strategy training by the instructors. Therefore, instructors would understand how to work with various learning styles of students and know what types of learning strategies should be taught to enhance students’ learning with the help of the explicit strategy training (Jie and Xiaoqing, 2006). The current study identified the preferred learning styles and learning strategies of distance learners in higher education. The effects of learning styles on learning strategies are still debated. Further research on this topic is therefore suggested.

### 5.1 Limitations

The present study has some limitations. First, the participants of the study were one of the limitations as the study only included students in distance education at a public university. This study was also limited to the learning strategies and the e-learning style scales for online environments. The self-reporting nature of the responses to the scale items during the data- gathering process may also be considered a limitation. It is suggested that future researchers conduct a sequential mixed-method to validate the findings of learners’ learning strategies and learning styles in the context of distance learning in higher education.

## Data Availability

The original contributions presented in the study are included in the article/supplementary material, further inquiries can be directed to the corresponding author/s.
